# An Intramuscular Injection of Mixed Testosterone Esters Does Not Acutely Enhance Strength and Power in Recreationally Active Young Men

**DOI:** 10.3389/fphys.2020.563620

**Published:** 2020-09-23

**Authors:** Sara Amalie Solheim, Jakob Mørkeberg, Anders Juul, Stine Yde Freiesleben, Emmie N. Upners, Yvette Dehnes, Nikolai Baastrup Nordsborg

**Affiliations:** ^1^Department of Nutrition, Exercise and Sports, University of Copenhagen, Copenhagen, Denmark; ^2^Anti Doping Denmark, Brøndby, Denmark; ^3^Department of Growth and Reproduction, Rigshospitalet, University of Copenhagen, Copenhagen, Denmark; ^4^Norwegian Doping Control Laboratory, Oslo University Hospital, Oslo, Norway

**Keywords:** testosterone, neuromuscular performance, jump height, rate of force development, maximal voluntary contraction, sprint performance

## Abstract

**Purpose**: Limited data are available on the acute performance-enhancing effects of single-dose administration of testosterone in healthy humans. Studies of testosterone administrations to healthy humans are rare due to the difficult nature and necessity of close clinical monitoring. However, our unique physiological experimental facilities combined with close endocrinological collaboration have allowed us to safely complete such a study. We tested the hypothesis that an intramuscular injection of 250 mg mixed testosterone esters (TEs) enhances physical performance in strength and power exercises acutely, measured 24 h after injection. Additionally, we investigated whether the basal serum testosterone concentration influences the performance in countermovement jump (CMJ), 30-s all out cycle sprint, and one-arm isometric elbow flexion.

**Methods**: In a randomized, double-blind, placebo-controlled design, 19 eugonadal men received either a TE (*n* = 9, 23 ± 1 years, 183 ± 7 cm, 83 ± 10 kg) or a PLA (*n* = 10, 25 ± 2 years, 186 ± 6 cm, 82 ± 14 kg) injection. Hormonal levels and the performance in CMJ, 30-s all out cycle sprint, and one-arm isometric elbow flexion were measured before and 24 h after injection.

**Results**: Firstly, an intramuscular injection of 250 mg mixed TEs did not enhance the vertical jump height in a CMJ test, peak power, mean power, and fatigue index in a 30-s all-out cycle sprint or rate of force development and maximal voluntary contraction in a one-arm isometric elbow flexion 24 h post-injection. Secondly, baseline testosterone levels appeared not to influence performance in strength and power exercises to a large extent in healthy, recreationally active young men.

**Conclusion**: A single intramuscular injection of 250 mg mixed TEs has no acute ergogenic effects on strength and power performance in recreationally active, young men. This novel information has implication for basic physiological understanding. Whether the same applies to an elite athlete population remains to be determined. If so, this would have implications for anti-doping efforts aiming to determine the most cost-efficient testing programs.

## Introduction

Long-term testosterone administration has well-known physiological effects such as inducing skeletal muscle hypertrophy (Griggs et al., 1989), accelerated lipolysis, and associated reduction of total body fat (Rebuffé-Scrive et al., 1991), as well as accelerated erythropoiesis (Beggs et al., 2014). Consequently, the anabolic androgenic steroid hormone testosterone and its synthetic analogues are some of the most widely used doping substances in both competitive sports (World Anti-Doping Agency, 2019; United States Anti-Doping Agency, 2020) and recreational exercise training (Sagoe et al., 2014). Moreover, long-term testosterone usage for nontherapeutic reasons can have adverse health consequences such as cardiomyopathy, dyslipidemia, and hypogonadism (Pope et al., 2014), and is thus not only regarded as a threat against fair sports but also a public health problem.

Short-term testosterone usage may also have ergogenic effects, illustrated by increased maximal bench press strength and total work in a 10-s cycle sprint in nine healthy, weight-trained, young men following 3 weeks with intramuscular injections of 200–300 mg/week testosterone enanthate, but not placebo (PLA), combined with heavy strength training (Rogerson et al., 2007). However, limited data are available on whether a single testosterone dosage induces acute ergogenic effects in humans. As extensively reviewed by Michels and Hoppe (2008) and Foradori et al. (2008), previous studies suggest that testosterone also has rapid, non-genomic actions (e.g., directly *via* ion channels and transporters or indirectly through second messengers) that can occur on a very short-term timescale (i.e., seconds, minutes, and hours) after testosterone stimulation. For example, testosterone stimulation produces a rapid increase in intracellular Ca^2+^ concentration in cultured rat myotubes within seconds to minutes (Estrada et al., 2003) and promotes insulin-like effects in incubated human skeletal muscle cells (Antinozzi et al., 2017), while stimulation with dihydrotestosterone, a product of testosterone metabolism, increases force production in intact isolated mice skeletal muscle fibers (Hamdi and Mutungi, 2010). Hence, acute testosterone administration can potentially stimulate augmented maximal voluntary contraction (MVC) force production and affect muscle energy metabolism in humans. Furthermore, increased plasma levels of testosterone, measured at 48 h post-administration of human chorionic gonadotropin, reportedly reduce the cortical motor threshold to evoke *m. interosseous dorsalis I* motor responses to transcranial magnet stimulation in healthy males (Bonifazi et al., 2004). This facilitates the corticospinal pathway (Bonifazi et al., 2004) that, in turn, can influence muscle activity and lead to faster muscle activation, which potentially can contribute to increased rate of force development (RFD) in voluntary movements. In that study, an intervention with human chorionic gonadotropin-stimulated endogenous testosterone production, rather than testosterone administration, was applied, which likely would delay the onset of action compared with an intramuscular injection of testosterone. Additionally, acute testosterone therapy causes vasodilatation (Pugh, 2003; Smith, 2008) and increased cardiac output *in vitro* (Smith, 2008) as well as in chronic heart failure patients (Pugh, 2003). Moreover, Carré et al. (2017) demonstrated a rapid increase in aggressive behavior within an hour, following a single testosterone gel administration in men with dominant or impulsive personality styles. This may confer psychological advantages in sports. Thus, based on the existing literature, it is possible that a testosterone injection induces acute (seconds to hours) performance-enhancing effects, which may provide testosterone users with an acute competitive edge if administering testosterone right before or during a competition in strength and power disciplines (e.g., weightlifting, powerlifting, jumping, and sprinting). However, until further research on humans is conducted, the effects of single-dose administration on human exercise performance remain speculative. If conducting such studies, measurable effects are most likely to occur at the time point where maximal pharmacological activity is expected. Based on plasma analysis following Sustanon® 250 administration, peak circulatory concentrations of the esters occur within 24–72 h of post-injection, with individual differences and depending on the length of the ester side chain (Forsdahl et al., 2015). Therefore, given the timeline of reported biological effects and pharmacokinetics, we tested the hypothesis that an intramuscular injection of 250 mg mixed testosterone esters (TEs; Sustanon®) enhances physical performance in countermovement jump (CMJ), 30-s all out cycle sprint, and one-arm isometric elbow flexion acutely 24 h after injection.

Noteworthy, positive relationships reportedly exist between natural serum testosterone levels at rest, vertical jump height (Bosco et al., 1996; Cardinale and Stone, 2006), and sprinting performance (Bosco et al., 1996) in elite athletes of various sports, which suggest that athletes’ performance capacities may be related to individual differences in basal testosterone levels. Thus, the magnitude of the effect of such single-dose testosterone injection may depend on the initial level of testosterone, causing a blunted biological effect in individuals with naturally high testosterone levels. Accordingly, we tested the hypothesis that the basal serum testosterone concentration influences the performance in CMJ, 30-s all out cycle sprint, and one-arm isometric elbow flexion in recreationally active men.

The evaluation of these questions would provide novel information for the basic physiological understanding of testosterone. Furthermore, knowledge about the performance-enhancing effects of a doping substance is important for anti-doping authorities. Whereas long-term testosterone administration increases the possibility for detection by anti-doping authorities and in private sports leagues falling outside of the World Anti-Doping Agency’s (WADA) jurisdiction, detection of short-term usage, or even a single dosage, requires more frequent sampling. This is associated with increased athlete discomfort as well as increased expenses related to sample collection and analysis. Hence, information about potential acute effects of testosterone is of high relevance when anti-doping authorities determine the most cost-efficient testing programs.

## Materials and Methods

### Subjects

Twenty-four recreationally active men volunteered to participate in the study. The sample size and power estimation were based on pilot experiments and similar experiments in the literature. The inclusion criteria were an age between 20 and 30 years and analytical values were within the population reference intervals for blood count, lipid profile, and liver function. The exclusion criteria were current or previous long-term heavy strength training, the presence of steroid abuse in a screening urine sample, contraindications for testosterone administration (i.e., prostate cancer, liver tumor, and breast carcinoma), and previous illness requiring hospitalization. The screening urine sample was analyzed according to the WADA guidelines at the Norwegian Doping Control Laboratory, Oslo University Hospital, Oslo, which is accredited by WADA and Norwegian accreditation (ISO/IEC 17025). Four participants were excluded after medical examination due to hemoglobin variant, factor V Leiden mutation, hypothyroidism, and previous testicular cancer, while one person dropped out. Characteristics of the 19 eugonadal male participants completing the study are presented in Table 1. The subjects’ health states were monitored throughout the experimental period. One participant was unable to complete the post-injection performance measurements due to illness and was, thus, only included in the testing of the second hypothesis. All subjects were fully informed, both orally and in writing, of the experimental procedures and of potential risks and discomforts associated with participation, before signing a written consent. The study was approved by the local ethics committee of Copenhagen, Denmark (H-17011319), and performed in accordance with the Declaration of Helsinki of 1964 and its later amendments.

**Table 1:** Subject characteristics.

	PLA	TE	Total
*n*	10	9	19
Age (years)	25 ± 2	23 ± 1	24 ± 2
Height (cm)	186 ± 6	183 ± 7	185 ± 7
Body weight (kg)	82 ± 14	83 ± 10	82 ± 12

### Study Design

The study was a randomized double-blind placebo-controlled trial and was part of a larger anti-doping research project that aimed to further develop analytical methods to detect doping with TEs (Solheim et al., 2020). For ethical reasons, we are highly focused on providing as much information as possible from the obtained data. In the present part, outcome measures of the physiological response to TE administration are presented. The subjects were randomly assigned to either a TE group or a PLA control group to evaluate the acute effect of TE administration on performance, while pooled baseline results for all participants were used to investigate the existence of a correlation between serum testosterone levels and performance capacities. As previously done (Chung et al., 2007; Forsdahl et al., 2015), the TE group received a single dose of 250 mg mixed TEs as 1 ml Sustanon® 250 (Aspen Pharma, Dublin, Ireland), a blend of four esterized testosterone compounds (30 mg testosterone propionate, 60 mg testosterone phenylpropionate, 60 mg testosterone isocaproate, and 100 mg testosterone decanoate). Sustanon® was applied because of the high relevance to doping control since the wash-out period is relatively short (2–3 weeks) and the product is known to be misused in athletic populations. Additionally, if study participants decide to leave the study, the wash-out period of Sustanon® is among the shortest of the available injectable oil-based products. The PLA control group was subjected to a sham injection of 1 ml saline. The intramuscular injection in *m. gluteus maximus* was performed on day 0 by a nonblinded medical doctor neither involved in the bioanalytical work nor the performance measurements.

### Exercise Protocol

**Figure 1:**
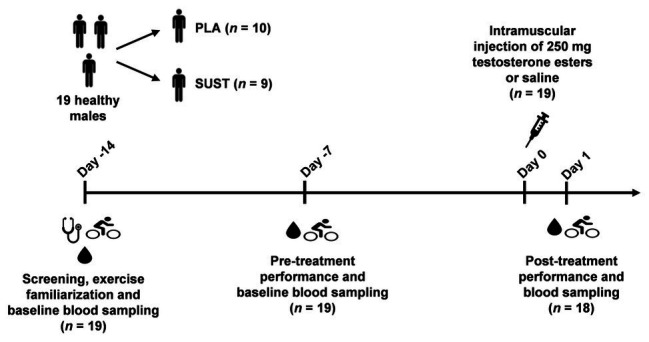
Timeline illustrating the procedure of the study. The number of participants is indicated in parentheses. PLA group, placebo group; TE group, testosterone ester group.

The study timeline is illustrated in Figure 1, and the testing was conducted over a 2-month period in the fall. Two weeks prior to the injection (Day-14), the subjects were well familiarized with the physical test protocol to reduce any learning effects. The familiarization protocol was identical with the pre-injection (Day-7) and post-injection (Day 1) protocol, which consisted of a 30-s all out sprint test, followed by a one-arm isometric elbow flexion test and a CMJ test. A 14.5 min recovery separated the 30-s all out sprint test and the warm-up to the one-arm isometric elbow flexion test, while the one-arm isometric elbow flexion test and the warm-up to the CMJ test were separated by 5 min recovery. The procedures for the performance tests are described in the following sections. Based on the timeline of reported biological effects and pharmacokinetics, the post-injection performance was evaluated 24 h after (Day 1) the injection, around the time point where maximal pharmacological activity was expected (Forsdahl et al., 2015). Serum testosterone concentrations vary throughout the day in a diurnal cycle (Piro et al., 1973; Albrethsen et al., 2020). Therefore, to minimize the influence of circadian hormonal changes on the performance measurements, the injections and testing sessions took place around the same time of the day. All sessions were completed between 7 AM and 3 PM, with a difference of 02:37 ± 01:36 (hh:mm) between each participant’s earliest and latest sessions and of 01:48 ± 01:22 (hh:mm) between each participant’s pre‐ and post-injection testing time points. To mimic a real-life situation, diet was not controlled prior to the tests but subjects were instructed to prepare, both physically and mentally, for “maximal performance” in the same matter on every test day. This involved no high-intensity/long-duration training the day before the tests, usual eating habits, no food intake from 2 h before test start, and being well-hydrated. Furthermore, participants were not allowed to consume coffee or other products containing caffeine before performance testing.

All performance measurements were carried out by the same blinded personnel. To evaluate whether the blinding procedure was successful, and whether the participants experienced any side effects, the subjects filled out a questionnaire following the post-injection tests.

#### 30-s All-Out Cycle Sprint Test

The sprint performance was evaluated by a 30-s all-out cycle sprint test. The test began with a warm-up for 6 min at 90 W followed by another 6 min at 150 W, on a mechanically-braked cycle ergometer (Monark 839E, Monark Exercise, Varberg, Sweden). Three minutes separated the warm-up and the test. The sprint performance was evaluated on a Peak Bike 894E cycle ergometer (Monark Exercise, Varberg, Sweden), with identical adjustments of saddle and handlebars within each subject. The test was initiated by subjects reaching >100 rpm during unloaded pedaling to overcome the flywheel inertia. Subsequently, the braking resistance of 0.1 kg per kg body mass was applied instantaneously and data collection commenced. The subjects were instructed not to pace themselves for a high average power output but to go all-out from the beginning and pedal as fast as possible for the following 30 s. Computer-based testing software (Monark Anaerobic Test Software v.1.0.15.0, Monark Exercise, Varberg, Sweden) recorded peak power (PP) as the highest running average of 1 s in W, mean power (MP) as the average power of the entire test in W, and fatigue index (FI) as the relative power decline from start to end (Ozkaya et al., 2018).

#### One-Arm Isometric Elbow Flexion Test

Maximal voluntary contraction and rate of force development were evaluated in an isometric elbow flexion test of the participants’ dominant arm. The warm-up consisted of 2 × 15 repetitions of dynamic elbow flexions with 3 kg resistance, followed by 2 × 5-s isometric contractions at 50% of MVC and one 5-s isometric contraction at 70% of MVC. The sets were separated by 30 s rest. The subjects performed the test in a diagonal half-kneeling position with their non-dominant arm placed behind their back. The dominant arm was supported at shoulder height and fixed with the forearm supinated and the elbow angle at 90°, in a custom-made apparatus coupled to a strain gauge load cell (Tedea-Huntleigh Model No. 615, 2006, Herzliya Pituach, Israel; Kilen et al., 2015). The subjects performed 3 × 5-s maximal contractions interspersed with 55 s rest. Visual real-time feedback on a computer screen and verbal encouragement were given to all participants during all contractions. The graphical visualization was implemented by LabChart software (PowerLab System v.8.1.5, ADInstruments, Oxford, United Kingdom). The subjects were explicitly told to reach peak force as quickly as possible by contracting the elbow flexors “hard and fast” and to keep the tension for 5 s. Contractions with uncontrolled pre-tension or countermovement were rejected (Maffiuletti et al., 2016), and the contraction procedure was repeated until the subjects had performed three valid contractions.

The force was sampled at 1,000 samples/s by a PowerLab 16/30 A/D converter (ADInstruments, Oxford, United Kingdom). The outcomes of the test were MVC and RFD. MVC was evaluated as the peak force (N) after smoothing the signal, while RFD (N/s) was defined as the peak slope of the Newton-time curve during 50-ms sampling windows in the original signal.

#### Countermovement Jump Test

The no-arm-swing version of the CMJ was used to assess jump height. Before the test, the subjects performed three submaximal jumps. When initiating the jump, the subjects stood in an upright position with their feet shoulder width apart and the toes pointing forward or slightly outward. The hands were placed on the hips and held there through the jump to eliminate the effect of arm swing on jump height (Domire and Challis, 2010). The subjects then performed the jump by bending the knees to approximately 90° and immediately reversing the movement to jump as high as possible. The subjects were instructed not to bend their knees during the jump and to land with flat feet. Each subject performed three maximal jumps separated by a 30-s recovery. Jumps with incorrect technique were rejected, and the jumping procedure was repeated until the subjects had performed three accepted jumps. A computer software (Optojump Next v.1.10.19.0, Microgate, Bolzano, Italy) estimated the jump height based on the flight time, which was measured by an optical time system (Optojump Next 3 cm, Microgate, Bolzano, Italy).

#### Test-Retest Reliability

Before the study, test-retest reliability of the CMJ test and the isometric elbow flexion test was measured in a volunteering, physically active group of five men and three women. The mean ± standard deviation (SD) age, height, and body weight of the group were 23 ± 3 years, 179 ± 10 cm, and 80.8 ± 12.8 kg, respectively. Each subject was tested in the same manner as in the intervention study, i.e., with a familiarization visit followed by 2 days of performance measurements at least 1 day apart. There were no significant differences in performance across the days of testing, neither for jump height 0.4 ± 1.9 cm (*p* = 0.540), RFD −51.8 ± 718.7 N/s (*p* = 0.841) nor for MVC 5.8 ± 30.7 N (*p* = 0.608). The coefficients of variation were 5.0% for the CMJ test, 18.6% for isometric one-arm elbow flexion RFD, and 7.7% isometric one-arm elbow flexion MVC. The test-retest reliability of the 30-s all-out cycle sprint test has recently been measured to be high for new generation power indices in 32 well-trained men, with coefficients of variation of 1.0% for peak power, 0.9% for mean power, and 2.8% for fatigue index (Ozkaya et al., 2018).

### Blood Sampling and Hormone Analyses

Venous blood samples for analysis of reproductive hormones were collected in the nonfasting state 14 and 7 days pre-administration (Day-14 and Day-7 in Figure 1) and 1 day post-administration (Day 1 in Figure 1) between 7 AM and 3 PM, with a difference of 1:48 ± 1:22 (hh:mm) between each participant’s sample collection time points. The venous blood samples were collected from an antecubital vein at least 2 h after the last workout and following at least 10 min of rest in a normal seated position with the feet on the floor. Blood samples were stored at 4°C and delivered for analysis at the Department of Growth and Reproduction, Rigshospitalet, Copenhagen, (DS/EN ISO 15189) within 2 h of collection or at the Hormone Laboratory, Oslo University Hospital, Oslo, (NS/EN ISO 17025) within 48 h of collection. Blood samples were clotted and centrifuged, and serum was stored at −20°C until hormone analyses were performed. Serum total testosterone at Day-7 and Day 1 was determined at the Hormone Laboratory, Oslo, by an inhouse LC-MS/MS method with a limit of quantification of 0.10 nmol/L and interassay coefficient of variation (CV) of 7% (Dahl et al., 2018). Serum from Day-14 and Day 1 was analyzed for luteinizing hormone (LH), follicle-stimulating hormone (FSH), oestradiol, and sex hormone binding globulin (SHBG) at Rigshospitalet, Copenhagen, by methods accredited by the Danish Accreditation Fund under the registration number 1013. Concentrations of LH and FSH were measured by time-resolved immunofluorometric assays (Delfia, Wallac Oy, Turku, Finland) with limits of detection (LOD) of 0.05 U/L and interassay CVs of ~2% in both gonadotropin assays. Oestradiol was measured by radioimmunoassay (Pantex, Santa Monica, US) with LOD of 18 pmol/and interassay CV of <13%. SHBG was measured using the *chemiluminescent* sandwich ELISA immunoassay (Access 2, Beckman Coulter, Co. Clare, Ireland) with interassay CV of ≤6% and LOD of 0.33 nmol/L.

### Statistics

SPSS was used for statistical analyses (IBM SPSS Statistics, version 25). Results are presented as the means ± SD. For the CMJ test and the isometric elbow flexion test in the intervention, data points that fell outside ±1 SD of the means of each subject’s accepted attempts were discarded from the dataset. A linear mixed model for repeated measures (Cnaan et al., 1997) was applied to determine if significant interactions or main effects existed for “time” (PRE, POST) and “treatment” (PLA, TE) on performance and reproductive hormones in the 18 subjects completing both the pre‐ and post-administration performance measurements. Repeated measures were identified by participant number, and Sidak adjustment was applied for pairwise comparisons. The level of statistical significance was set at *p* < 0.05.

The between-test CV and one-way repeated-measures analysis of variance, with “time” (i.e., familiarization test, test 1 and test 2) as the within-subject factor, were used to evaluate test-retest reliability of the CMJ test and the isometric elbow flexion test in a group of eight subjects (*n* = 8). The relative CV was calculated by dividing the SD of the difference in test-retest results (*σ*) by the grand mean (*x̄*) and multiplying the quotient by 100%: %CV = (*σ*/*x̄*) × 100%.

The effectiveness of blinding in each administration group was evaluated by the Bang blinding index (BI; Bang et al., 2004) using the questionnaire results from the 18 subjects completing both the pre‐ and post-administration performance measurements. The Bang BI was calculated as BI_1_ = *P*_1|1_ – *P*_2|1_ and BI_2_ = *P*_2|2_ – *P*_1|2_ for the TE group and the PLA control group, respectively. *P*_j|i_ = *P*_(guess *j*|assigned administration *i*)_ represents the conditional probability, where *i* = 1 (TE), 2 (PLA) and *j* = 1 (TE), 2 (PLA). The index scores were used to calculate the variance as *var*(BI*_i_*) = {(*P*_1|*i*_ (1-*P*_1|*i*_) + *P*_2|*i*_ (1-*P*_2|*i*_) + 2*P*_1|*i*_*P*_2|*i*_)}/*n_i_*, and the 95% CI as BI*_i_* ± 1.96 × √*var*(BI*_i_*). The Bang BI ranges from −1 to 1, where −1 indicates that all subjects guessed the incorrect administration, 0 indicates that all subjects randomly guessed, and 1 indicates that all subjects guessed the correct administration. If CI included the null value, administration-arm blinding had been maintained. Participants’ conjectures about the administration allocation were compared for the TE group and PLA control group using the likelihood ratio (*χ*^2^).

Correlations between pooled basal serum levels of testosterone and performance measurements (*n* = 19) were assessed using Pearson’s correlation coefficients and interpreted as trivial for *R* = 0.0–0.1, small for *R* = 0.1–0.3, moderate for *R* = 0.3–0.5, large for *R* = 0.5–0.7, and very large for *R* = 0.7–0.9 (Hopkins, 2002).

## Results

### Testosterone Ester Injection and Acute Performance

**Figure 2:**
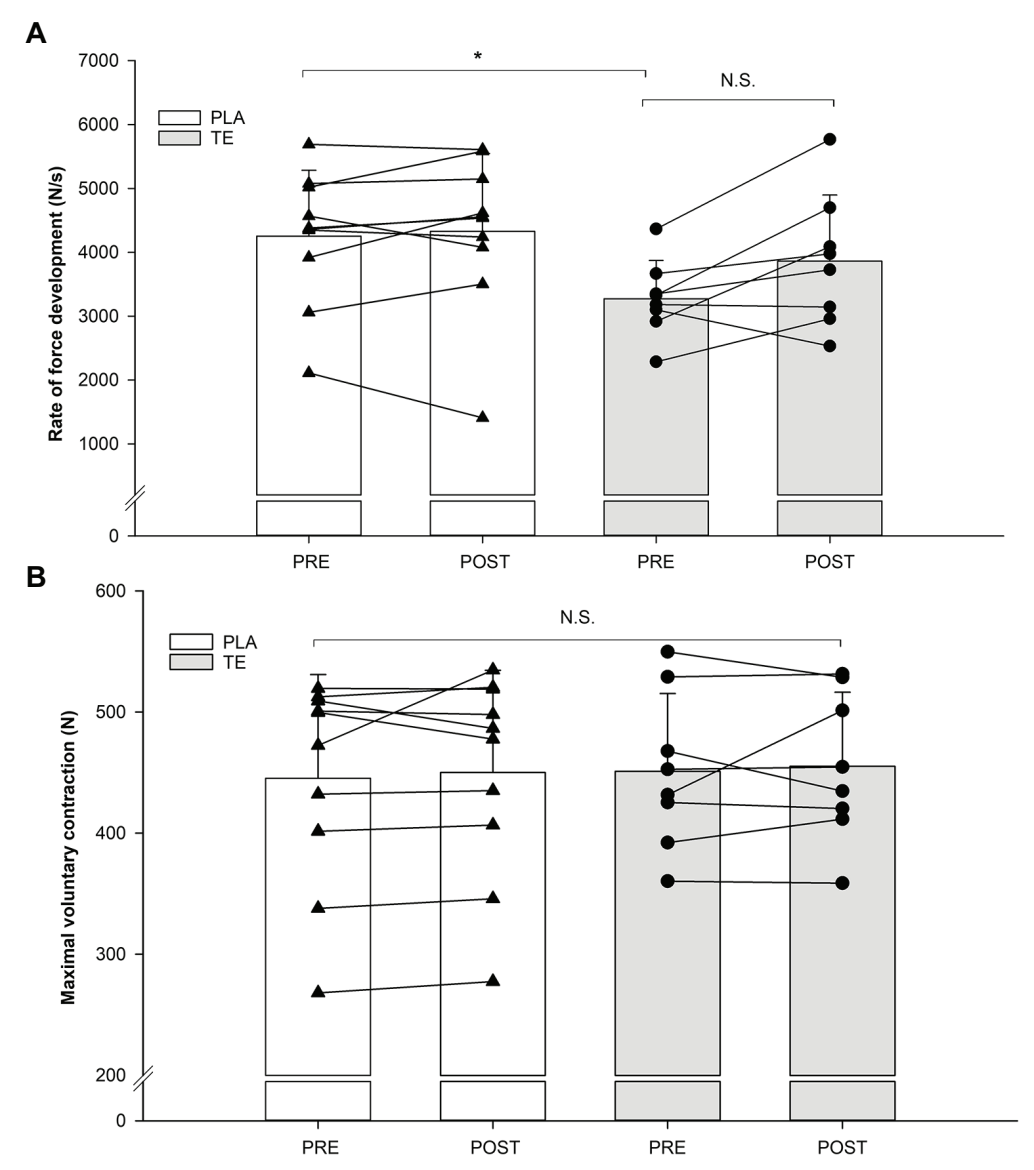
Individual values of rate of force development **(A)** and maximal voluntary contraction **(B)** in a one-arm isometric elbow flexion before (pre) and after (post) placebo (PLA; *n* = 10) administration (white) and testosterone ester (TE; *n* = 8) administration (gray). *Histograms* indicate means with error bars indicating one standard deviation of the mean. ^*^Significant (*p* < 0.05) main effect of treatment. NS, not significant.

A main effect existed for treatment (*p* = 0.04) for the RFD measurements. The *post hoc* analysis revealed that there was a significant difference (*p* = 0.03) between the initial group means in RFD, but no significant changes within treatment (Figure 2). No main effects existed for the MVC measurements (Figure 2).

No main effect of time, treatment, or time × treatment was observed for PP, MP, or FI in the sprint test (*p* > 0.05; Table 2). The non-significant changes from before to after injection was 0.9 ± 3.6% (*p* = 0.93) for the TE group and −0.9 ± 2.5% (*p* = 0.92) for the PLA control group in peak power, 0.1 ± 1.5% (*p* = 1.00) for the TE group and 0.2 ± 2.5% (*p* = 0.99) for the PLA control group in mean power, and 1.5 ± 3.9% (*p* = 0.80) for the TE group and 1.4 ± 8.4% (*p* = 0.80) for the PLA control group in fatigue index.

**Table 2:** Sprint performance.

Variable	Treatment	Pre	Post
Peak power (W)	PLA	1,124 ± 186	1,115 ± 197
	TE	1,110 ± 186	1,119 ± 182
Mean power (W)	PLA	815 ± 109	816 ± 104
	TE	811 ± 109	811 ± 106
Fatigue index (%)	PLA	52 ± 5	52 ± 6
	TE	50 ± 6	51 ± 6

Performance outcomes (mean ± SD) for the 30-s all-out cycle sprint test before (pre) and after (post) placebo (PLA) and testosterone ester (TE) administration.

No significant main effects existed for jump height, which remained similar before and after the injection for both the TE group (Pre: 34.0 ± 5.4 cm; Post: 33.9 ± 5.5 cm, *p* = 0.97) and the PLA control group (Pre: 35.0 ± 6.5 cm; Post: 35.6 ± 5.5 cm, *p* = 0.83).

### Testosterone Ester Injection and Reproductive Hormones

**Figure 3:**
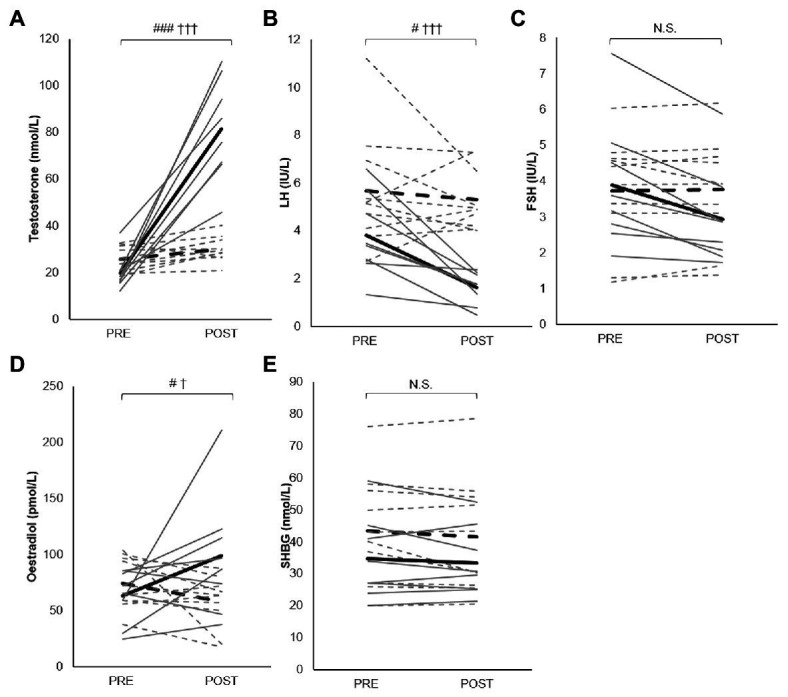
Changes in levels of serum testosterone **(A)**, luteinizing hormone (LH; **B**), follicle stimulating hormone (FSH; **C**), oestradiol **(D)**, and sex hormone binding globulin (SHBG; **E**) from pre to post TE administration (solid lines; *n* = 8) and PLA administration (dashed lines; *n* = 10). Gray lines indicate individual changes while black lines indicate group mean changes. Statistically significant differences are: ^#^*p* < 0.05 compared with pre-injection, ^###^*p* < 0.001 compared with pre-injection, ^†^*p* < 0.05 compared with PLA, ^†††^*p* < 0.001 compared with PLA.

There were main effects of time (*p* < 0.001), treatment (*p* < 0.001), and a time × treatment interaction (*p* < 0.001) for serum testosterone, main effects of time (*p* = 0.03) and treatment (*p* < 0.001) for LH, and a time × treatment interaction (*p* = 0.03) for oestradiol when the effect of TE injection was investigated (Figure 3). The *post hoc* analysis demonstrated that serum testosterone was elevated (*p* < 0.001) from pre-administration (19.8 ± 7.6 nmol/L) to post-administration (81.4 ± 21.9 nmol/L) in the TE group and that the post-administration concentration was higher (*p* < 0.001) in the TE group (81.4 ± 21.9 nmol/L) compared with in the PLA control group (30.0 ± 5.4 nmol/L). Furthermore, LH was reduced (*p* = 0.02) from pre (3.8 ± 1.7 IU/L) to post-administration (1.6 ± 0.7 IU/L) in the TE group, and lower (*p* < 0.001) post-administration in the TE group (1.6 ± 0.7 IU/L) compared with in the PLA control group (5.3 ± 1.3 IU/L). The oestradiol concentration was elevated in the TE group (99.0 ± 54.2 pmol/L) compared with pre-administration (63.5 ± 24.2 pmol/L, *p* = 0.04) and compared with the placebo (58.0 ± 23.2 pmol/L, *p* = 0.05). No main effect of time, treatment, or time × treatment existed for neither FSH nor SHBG.

### Testosterone Ester Injection: Blinding and Side Effects

The subjects were generally unaware of the administration they received. The BI was 0.00 (95% CI: −0.49, 0.49) in the TE group and 0.20 (95% CI: −0.34, 0.74) in the PLA control group. The two proportions of blinding were not significantly different [*χ*^2^ (2) = 1.99, *p* = 0.37]. Concerning side effects, seven out of eight in the TE group experienced discomfort at the site of injection, while the corresponding proportion in the PLA control group was one out of 10.

### Resting Serum Testosterone Levels and Performance

**Figure 4:**
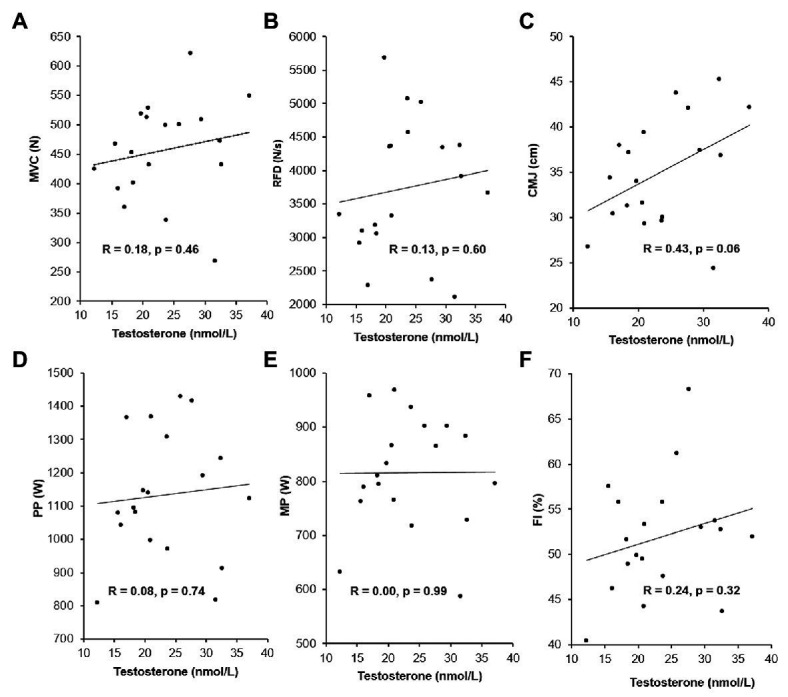
Relationships between serum testosterone concentrations at rest and baseline performance measures (*n* = 19). Maximal voluntary contraction (MVC; **A**), rate of force development (RFD; **B**), counter movement jump height (CMJ; **C**), peak power (PP; **D**), mean power (MP; **E**), and fatigue index (FI; **F**). The Pearson’s correlation coefficients, R, and values of *p* are illustrated in chart.

The subjects’ pooled serum testosterone level at baseline (23.3 ± 6.9 nmol/L) was in the midnormal range for healthy young men. The correlations between the combined TE and PLA groups resting serum testosterone concentration and performance measures at baseline are presented in Figure 4. None of the correlations were significant (*p* > 0.05). For MVC, RFD, PP, FI, and MP, there were trivial to small positive, non-significant correlations with resting testosterone levels, while there was a moderate positive, non-significant correlation for CMJ with resting testosterone levels.

## Discussion

Herein, we evaluated the acute effect of a single intramuscular injection of TEs on human physical performance in strength and power exercises. Further, we investigated if the basal serum testosterone concentration influences strength and power performance. If so, the biological effects of such single-dose administration could depend on the initial serum testosterone level. The major findings were 2-fold. Firstly, an intramuscular injection of 250 mg mixed TEs did neither enhance performance acutely in a CMJ test, a one-arm isometric elbow flexion test, nor a 30-s cycle sprint test in recreationally active men. Secondly, baseline testosterone levels appeared not to influence performance in strength and power exercises in healthy, recreationally active young men. The following sections discuss these findings in relation to the existing literature.

### Testosterone Ester Injection

Based on rodent studies and *in vitro* studies, it appears possible that testosterone has acute ergogenic effects on strength and power exercises in healthy humans. However, this has never been investigated in humans. This was thus the purpose and novelty of the present study. In contrast to the initial hypothesis, an intramuscular injection of 250 mg mixed TEs did neither enhance the vertical jump height in a CMJ test, PP, MP, and FI in a 30-s all-out cycle sprint, nor RFD and MVC in a one-arm isometric elbow flexion 24 h post-injection, which clearly demonstrate that the single TE injection has no acute ergogenic effects on human strength and power performance in recreationally active, young men. Thus, the potential testosterone-induced facilitation of the corticospinal pathway (Bonifazi et al., 2004), increase in cardiorespiratory capacity (Pugh, 2003; Smith, 2008), and augmentation in muscle calcium release (Estrada et al., 2003) and in muscle glucose metabolism (Antinozzi et al., 2017), if present, did not reach a level sufficient to affect human performance in the present study. In this regards, it must be stressed that some of the factors determining the performance in a 30-s all out sprint measured as mean power and fatigue index, e.g., the buffer capacity and muscle glycogen content, would likely not be affected only 24 h post-injection of an intramuscular testosterone injection. However, the potential acute rise in aggression after testosterone administration (Carré et al., 2017), in combination with the potential effect of testosterone on skeletal muscle activation and contractility (Estrada et al., 2003; Bonifazi et al., 2004), could acutely improve peak power output during a 30-s all-out sprint. Likewise, since lower-body RFD and maximal force production reportedly are the main contributors to vertical jump height (McLellan et al., 2011; McErlain-Naylor et al., 2014), testosterone administration could potentially increase CMJ height acutely by augmenting maximal muscle force production (Estrada et al., 2003; Hamdi and Mutungi, 2010) and by inducing more rapid muscle activation through facilitated corticospinal pathway (Bonifazi et al., 2004). However, testosterone-induced physiological changes may not always result in measurable alterations of human performance. In support of this notion, the maximal evoked *m. interosseus dorsalis I* response to cortical and cervical magnetic stimulation was similar before and 48 h after administration with 5,000 IU of human chorionic gonadotropin, despite at significantly reduced cortical motor threshold (Bonifazi et al., 2004).

The effects of testosterone administration on muscle strength, leg power (Bhasin et al., 2001; Storer et al., 2003), and muscle size (Bhasin et al., 2001) in healthy, young men are dose‐ and serum concentration dependent. Given that testosterone administration causes a feedback inhibition of LH and FSH (Schulte-Beerbühl and Nieschlag, 1980) and, thus, on endogenous testosterone production, the administered dose has to more than compensate for the drop in endogenous testosterone levels. In the present study, serum concentrations of LH were significantly reduced post-administration in the TE group, illustrating that a negative feedback loop was activated. Failure to observe altered performance may thus be explained by an insufficient increase in testosterone levels to induce measurable increases in strength and power. This seems likely to have been the case in the study by Crist et al. (1983), who found no consistent administration effect on lower or upper body isokinetic strength in men treated with either 100 mg/week testosterone cypionate, 100 mg/week nandrolone decanoate, or PLA for 3 weeks in a double-blind PLA-controlled design. Unfortunately, no hormone measures were reported. When comparing weekly injections of either 25 (*n* = 12), 50 (*n* = 12), 125 (*n* = 12), 300 (*n* = 12), or 600 (*n* = 13) mg of testosterone enanthate for 20 weeks in a double-blind design, a dose-relationship was apparent with significantly larger changes in leg press strength in the 300 and 600 mg/week groups than in the other groups (Bhasin et al., 2001; Storer et al., 2003). Moreover, in a double-blind PLA-controlled study, 3 weeks with intramuscular injections of 200–300 mg/week testosterone enanthate, combined with heavy strength training, was sufficient to increase maximal strength and 10-s cycle sprint performance significantly in healthy, young men (Rogerson et al., 2007). In the current study, a significant 3-fold elevation in mean serum testosterone concentration was observed from pre-administration to post-administration in the TE group, while it remained similar in the PLA control group. This indicates that the applied administration regime indeed elevated serum testosterone to levels outside the normal physiological range. Accordingly, serum oestradiol concentration was significantly elevated only in the TE group following administration (Figure 3), likely due to peripheral conversation of injected testosterone by aromatization (Matsumine et al., 1986). Additionally, the level of SHBG, which regulates the testosterone bioavailability, remained similar pre-administration to post-administration (Figure 3). Taken together, these observations suggest that the administration in the present study was sufficient to increase the concentration of unbound, bioavailable testosterone to supraphysiological levels in the treated subjects, which likely would have elicited measurable changes in the performance measures if acute ergogenic effects of testosterone existed.

This novel information has implication for the basic physiological understanding of how testosterone administration interacts acutely with exercise capacity and in relation to anti-doping efforts. From an anti-doping perspective, it is of outmost importance to intelligently target the right athlete at the right time to make best use of the available resources. In that regard, it is of interest that a single intramuscular injection of 250 mg mixed TEs appears not to enhance performance acutely in recreationally active, young men, at a time point where its use is likely to be detectable (Forsdahl et al., 2015). It must be noted that the findings in the present study are specific to the cohort under investigation, and it is unknown whether the results are applicable to females and elite athletes. For example, on the one hand, it could be speculated that the potential acute rise in aggression after testosterone administration is more likely to have a psychological effect on the performance in elite athletes, who are more used to doing all-out efforts than recreationally active individuals. On the other hand, testosterone gel administration reportedly potentiates aggressive behavior only in individuals scoring high on trait dominance or low on trait self-control (Carré et al., 2017), which suggests that a potential psychological effect on performance only would be apparent in individuals with dominant or impulsive personality styles. Notably, despite ranging from untrained to moderately trained individuals, the present relative pooled baseline MP result (9.9 W/kg) is in line with that reported for elite level (>9.8 W/kg) of large population of athletes (Zupan et al., 2009). If the results can be extrapolated to an elite athlete population, this suggests that athletes are likely not to have a competitive edge by abusing a single dose of TEs immediately before or during a competition in strength and power sports. Contrarily, it has previously been shown that consecutive administrations of testosterone over a period of several weeks enhance strength and power (Bhasin et al., 1996, 2001; Giorgi et al., 1999; Storer et al., 2003; Rogerson et al., 2007). This implies that in order for an athlete to improve performance through testosterone doping, administrations are likely to occur out of competition, e.g., in the lead up to competitions. Having that said, a single-dose administration of testosterone is still a violation of the WADA rules and possible adverse health consequences cannot be ruled out.

It may be questioned whether the applied dose is of high relevance as athletes’ steroid regimens are found to range from 250 to more than 2,500 mg/week (Evans, 1997; Yu et al., 2014). However, anecdotal evidence suggests that athletes tend to “microdose” doping to reduce the risk of being caught. This may also be the case for testosterone and its synthetic analogues, either by injections, gel, patches, or oral capsules. Worth mentioning is that the selection of the 250 mg dose in the present intervention was based on the ethical consideration that this has previously been safely administered to men in controlled studies (Chung et al., 2007; Forsdahl et al., 2015).

### Resting Serum Testosterone Levels

Hormonal profiles vary with age, sex, and physique (Healy et al., 2014) but relationship between testosterone level and athletic performance has not yet been fully explored. Despite varying testosterone levels (12.2–37.1 nmol/L), and opposed to our initial hypothesis, basal serum levels of testosterone did not predict the performance in CMJ, 30-s all out cycle sprint, or one-arm isometric elbow flexion in healthy, recreationally active young men (Figure 4).

Our findings contradict the significant positive relationships between natural serum testosterone levels and vertical jump height (Bosco et al., 1996; Cardinale and Stone, 2006) and sprinting performance (Bosco et al., 1996) in elite athletes. The discrepancy may be related to the inclusion of a mixed group of subjects in the present study in regards to training background and existing strength level, because resting salivary testosterone levels are found to predict performance outcomes only in individuals with high strength levels (Crewther et al., 2012). However, if dividing the subjects into two groups based on their baseline MVC performance, <450 N (*n* = 8, MVC: 381.1 ± 57.0 N) and >450 N (*n* = 11, MVC: 512.2 ± 45.8 N), testosterone levels cannot account for the difference (*p* < 0.001) in mean performance, evident by both similar testosterone levels (21.6 ± 7.3 nmol/L in the <450 N group vs. 24.6 ± 6.5 N in the >450 N, *p* = 0.346) and non-significant moderate correlations for MVC and testosterone (<450 N: *r* = −0.39, *p* = 0.334 and >450 N: *r* = 0.41, *p* = 0.210). This is supported by Crewther et al. (2016), who found no correlation between salivary testosterone concentrations and neuromuscular performance in neither elites nor non-elites. As an important note with regards to the study design in the present study, the relatively small sample size, males only and no elite athletes precludes firm conclusions regarding the correlation between serum testosterone levels and strength and power performances. However, combined with the findings by Crewther et al. (2016), our results suggest that testosterone alone does not predict performance. In support of this notion, sex differences in performance were explained by differences in lean body mass rather than serum testosterone levels in a large population of 454 male and 239 female elite athletes in 15 competition categories (Healy et al., 2014).

The present findings add to the basic physiological understanding of testosterone. From an anti-doping perspective, the lack of strong correlations between basal serum testosterone concentration and performance capacities suggests that the biological effect of a single intramuscular testosterone injection is not dependent on the initial level of serum testosterone. Thus, the initial testosterone concentration is likely not to cause a blunted biological effect of a single-dose administration in individuals with naturally high testosterone levels.

## Conclusion

An intramuscular injection of 250 mg mixed TEs does not enhance performance acutely when evaluated as a CMJ, a maximal one-arm isometric elbow flexion, and a 30 s all-out cycle sprinting in recreationally active, young men. Potential delayed effects were not evaluated. Additionally, strong correlations between performance in strength and power exercises and basal serum testosterone levels are not evident in a group of healthy, recreationally active young men.

Because this is the first study to investigate the acute ergogenic effects of testosterone administration on human physical performance, the results provide new insight into the basic understanding of the performance-enhancing effects of testosterone. Whether the findings apply to females and an elite athlete population remains to be determined.

## Data Availability Statement

The raw data supporting the conclusions of this article will be made available by the authors, without undue reservation.

## Ethics Statement

The studies involving human participants were reviewed and approved by the local Ethics Committees of Copenhagen. The patients/participants provided their written informed consent to participate in this study.

## Author Contributions

SS, SF, JM, AJ, YD, and NN conceived and designed the study. SS, SF, and EU conducted experiments. SS, SF, AJ, and YD analyzed data. SS and NN drafted the manuscript. All authors engaged in revising the manuscript and approved the final version of the manuscript.

### Conflict of Interest

SS and JM were employed by Anti Doping Denmark during the intervention and the preparation of this manuscript.

The remaining authors declare that the research was conducted in the absence of any commercial or financial relationships that could be construed as a potential conflict of interest

## Abbreviations

BI: Blinding index
CMJ: Countermovement jump
CV: Coefficient of variation
FI: Fatigue index
FSH: Follicle-stimulating hormone
LH: Luteinizing hormone
LOD: Limit of detection
MP: Mean power
MVC: Maximal voluntary contraction force
PLA: Placebo
PP: Peak power
RFD: Rate of force development
SD: Standard deviation
SHBG: Sex hormone binding globulin
TE: Testosterone ester
WADA: World Anti-Doping Agency
